# The nutrition transition in Colombia over a decade: a novel household classification system of anthropometric measures

**DOI:** 10.1186/s13690-014-0057-5

**Published:** 2015-02-16

**Authors:** Diana C Parra, Lora Iannotti, Luis F Gomez, Helena Pachón, Debra Haire-Joshu, Olga L Sarmiento, Anne Sebert Kuhlmann, Ross C Brownson

**Affiliations:** Program in Physical Therapy, School of Medicine, Washington University in St. Louis, St. Louis, MO USA; George Warren Brown School of Social Work, Institute for Public Health, Washington University in St. Louis, St. Louis, MO USA; Departamento de Medicina Preventiva y Social. Facultad de Medicina, Pontificia Universidad Javeriana, Bogotá, Colombia; Food Fortification Initiative, Atlanta, USA; Hubert Department of Global Health, Rollins School of Public Health, Emory University, Atlanta, USA; Center for Diabetes Translation Research and Center for Obesity Prevention & Policy Research, Washington University in St. Louis, St. Louis, MO USA; Department of Public Health, School of Medicine, Universidad de los Andes, Bogotá, Colombia; Prevention Research Center in St. Louis, Brown School, Washington University in St. Louis, St. Louis, MO USA; Division of Public Health Sciences and Alvin J. Siteman Cancer Center, Washington University in St. Louis School of Medicine, St. Louis, MO USA

**Keywords:** Overweight, Obesity, Nutrition transition, Stunting, Dual burden, Malnutrition, Colombia

## Abstract

**Background:**

Overweight and underweight increase the risk of metabolic impairments and chronic disease. Interventions at the household level require the diagnosis of nutritional status among family members. The aim of this study was to describe the prevalence and patterns of various anthropometric typologies over a decade in Colombia using a novel approach that considers all children in the household as well as the mother. This approach also allows identifying a dual burden of malnutrition within a household, where one child may be overweight and another one undernourished.

**Methods:**

This study used data from the Demographic and Health Survey and the Colombian National Nutrition Survey [2000 n = 2,876, 2005 n = 8,598, and 2010 n = 11,349].

Four mutually exclusive household (HH) anthropometric typologies - *normal, undernourished, overweight/obese,* and *dual burden* - were created. Anthropometric information of height-for-age Z-scores (HAZ) and body-mass-index-for-age Z-scores (BMIz) in children under the age of 5 y, and on body mass index (BMI) in mothers, 18–49 y was used.

**Results:**

Prevalence of overweight/obese HHs increased between 2000 (38.2%) and 2010 (43.1%) (*p* < 0.05), while undernourished and dual burden HHs significantly decreased between 2005 (13.7% and 10.6%, respectively) and 2010 (3.5% and 5.1%, respectively) (*p* < 0.05). A greater increase of overweight/obesity was observed for the lowest quintile of wealth index (WI), with an increase of almost 10% between 2000 and 2010, compared to 2% and 4% for the fourth and highest WI, respectively. Although in 2010 there is still a higher prevalence of overweight/obesity HHs in urban areas (43.7%), the prevalence of overweight/obesity HHs in rural areas increased sharply between 2000 (34.3%) and 2010 (41.6%) (*p* < 0.05).

**Conclusion:**

The observed prevalence of dual burden households was not different from the expected prevalence. Results from this study indicate that although overweight/obesity continues to be more prevalent among high-income Colombian households, it is growing at a faster pace among the most economically disadvantaged.

**Electronic supplementary material:**

The online version of this article (doi:10.1186/s13690-014-0057-5) contains supplementary material, which is available to authorized users.

## Background

Adult obesity is ranked among the top ten leading attributable risk factors of mortality in Latin American countries [1]. Similarly, undernutrition is the leading mortality risk factor in children ages under 1 y in this region [1]. Despite worldwide improvement in life expectancy and reduction of child mortality, low birth-weight and childhood undernutrition continue to be significant problems in many countries from Latin America. Undernutrition has a negative impact on human capital and can lead to permanent and irreversible consequences that affect future generations [2,3]. Low birth weight and childhood undernutrition also increase the risk of developing obesity and co-morbidities in later life including metabolic syndrome, diabetes, and cardiovascular disease [4,5]. These risks are exacerbated by exposure to the increasingly prevalent obesogenic environment of countries undergoing rapid nutrition transition [6]. Colombia is experiencing a nutrition transition and has seen a steady increase in the average body mass index (BMI) of its population throughout the years; while at the same time childhood chronic undernutrition still persists, in both urban and rural areas [7].

Both underweight and overweight have devastating consequences for future health affecting the individual and society [2-5]. In the midst of this transition there is also an apparent paradox where undernutrition (e.g. based on anthropometry, biomarkers) and overnutrition (e.g. based on anthropometry) coexist within the same community and sometimes even within the same household or individual (i.e. a stunted child who is also overweight) [6,8-17]. This phenomenon is known as the dual burden of malnutrition [18,19], which has been operationalized in various ways. Some studies have defined the dual burden household as the presence of underweight and overweight members within the same home [17]. Other studies have focused exclusively on the coexistence of childhood chronic undernutrition, typically among children 5 y of age and younger, and overweight among mothers, particularly focusing on the youngest child-mother pair also known as stunted child – overweight mother (SCOM) [10]. In addition, most studies have focused on a particular region within a country or have only looked at national averages, not considering within-country differences and disparities. For instance, in Colombia, earlier research has shown that the national prevalence of SCOM was 5% in 1995 [10], but regional variations within Colombia have not been examined and large disparities with higher prevalence among the most economically disadvantaged groups are anticipated. Given concomitant high rates of childhood undernutrition and maternal overweight/obesity in specific regions of the country (i.e. La Guajira, a northern state bordered by the Caribbean sea), vast differences in the prevalence of dual burden are expected [7]. Another study exploring the specific phenomenon of the dual burden of malnutrition (an underweight and an overweight member in the same household) is from the Antioquia state of Colombia. This study included 1,699 homes and documented the prevalence of dual burden to be as high as 12%, with no significant differences between urban and rural areas [20].

A growing prevalence of overweight among disadvantaged populations has been reported in Brazil and Mexico [21-23]. It is likely that Colombia is following the same trend, but detailed information about this transition, including the dual burden of malnutrition taking into account regional differences is lacking. The last two decades in Colombia have implied profound economic, political and social transformations that have a direct effect on the nutritional status of the population. For instance, as a result of the Trade Agreement (TPA) with the United States (U.S.-Colombia Trade Agreement Now In Force, 2012), a greater access to highly processed foods, which has been found to be associated with obesity [24], is expected in the near future. It is likely that traditional diets and other cultural aspects related to eating habits have changed, as it has been the case in other countries from Latin America (Clark, Hawkes, Murphy, Hansen-Kuhn, & Wallinga, 2012, Hawkes & Thow, 2008) [25,26]. In addition, classification systems for anthropometric measures at the household level have been limited to only one child or specific members of the household. They also have not taken into account the possibility of concomitant stunting and overweight/obesity within one child, or all children in the household, or both; a recent phenomenon reported in countries undergoing nutrition transition [27,28]. It is important to document the current state of malnutrition problems in Colombia in particular among the most vulnerable groups including low income, rural populations, indigenous households, women and children.

Using data from the 2000, 2005, and 2010 Colombian Demographic and Health Survey (ENDS) and 2005–2010 National Nutrition Survey (ENSIN), which were conducted concomitantly in the years 2005 and 2010 [29-33], this study aims to document the changing patterns and prevalence of various anthropometric typologies at the household level, namely, normal, undernourished, overweight/obese, and dual burden. In addition, this study aims to document inequalities in the prevalence of these typologies by area of residence (rural versus urban) and wealth index using a novel household classification system of anthropometric measures. This study also explores regional differences by state, known as *departamentos* in Colombia.

## Methods

### Study design

We conducted a secondary data analysis of three cross-sectional, nationally representative samples (2000, 2005 and 2010) from Colombia [29-33]. In 2005 and 2010 we merged ENDS/ENSIN. Both of these surveys, ESIN and ENDS, interviewed the same households and the fieldwork was completed at the same time. The socio-demographic variables come from ENDS and the anthropometric measurements come from ENSIN. The surveys used a probabilistic, stratified, multistage, cross-sectional survey to obtain national and sub-regional representativeness (16 sub-regions), with oversampling of rural areas and low socioeconomic status (SES) groups. In 2000, 2005 and 2010, interviewers, mainly nutritionists, were selected to conduct anthropometric measurements following a rigorous standardized training averaging at least 5 weeks [29-33]. ENDS 2005 and 2010 included 99% of the urban and rural population. ENDS 2000 covered 98% of the national population as it excluded rural population from the San Andrés, Amazonas and Orinoco regions, due to difficulty in accessibility and high operational costs. Response rates for anthropometry were 93% in 2000 74% in 2005, and 85% in 2010 [29-33].

### Data sets and sampling

Households were used as the unit of analysis using individual measures to create the HH typologies, after merging relevant information from separate ENDS/ENSIN data sets (individual and household) [34]. For the purpose of the ENDS/ENSIN, a household is defined as a group of individuals consuming from the same food pot. Children with implausible values for weight, height and BMI (> −/+5 SD away from the median of the WHO reference population) [35] were excluded, going from 4,670 children to 4418 in 2000, from 14,597 to 14,088 in 2005, and from 18,578 to 18,101 in 2010. Eligible HHs were those where there was at least one child <5 y and their mother between 18 and 49 y; if there was more than one eligible case (i.e. more than one mother living in the household with children less than 5 y) the entire household was excluded. These households were excluded on the basis that multi-family or extended family homes might look very different from nuclear or traditional families, it would have also impede us from using our classification system since it is limited to the information of one mother and their children. Multi-family homes represented (4% or 136 household of the eligible sample in 2000, 6% or 664 households in 2005, and 8.6% or 945 households in 2010. In 2000, out of 3,346 eligible HHs, 2,876 (86%) were included in the analysis. In 2005, out of 10,939 eligible households, 8,598 (78.5%) were included in the analysis. In 2010, out of 14,323 eligible HHs, 11,349 (79.2%) were included in the analysis. Institutional Review Board approval for the analyses conducted in this study was not deemed necessary since there were no personal identifiers linking the data to individuals.

### Measures and variables

During ENDS/ENSIN [29-33] weight and height were measured with standardized equipment. Weight was measured to the nearest 0.1 kg using a digital weighing scale (SECA model 770; Brooklyn, NY), participants were instructed to wear light clothing and remove shoes. Height was measured to the nearest 1.0 cm with a portable stadiometer (Shorr Productions, Onley, MD) and length was measured in children 2 y or younger in a prone position, following established protocols [35,36].

#### Outcome variable

Four mutually exclusive categories describing household anthropometric typologies were developed based on children’s and maternal nutritional status (Table 1).

**Table 1:** **Household anthropometric typologies (Mutually exclusive categories)**

**Household anthropometric typology**	**Children’s anthropometric status**	**Mother’s BMI**
**Normal**	No stunting or obesity among any of the children	Normal BMI 18.5 – 24.9 kg/m^2^
**Undernourished**	At least one child is stunted (HAZ < −2) and the remaining children are either stunted or normal	Underweight BMI <18.5 kg/m^2^ or Normal BMI 18.5 – 24.9 kg/m^2^
**Overweight/obese**	At least one child is overweight/obese (BMIz > 2SD) and the remaining children are either overweight/obese or normal	Normal BMI 18.5 – 24.9 kg/m^2^ or Overweight/obese BMI ≥ 25 kg/m^2^
**Dual Burden**	At least one child is stunted (HAZ < −2) and the remaining children can be either stunted, normal, overweight/obese, or stunted and overweight/obese	Overweight/obese BMI ≥ 25 kg/m^2^
	At least one child is overweight/obese (BMIz > 2SD) and the remaining children can be either normal, overweight/obese, or stunted and overweight/obese	Underweight BMI <18.5 kg/m^2^

Nutritional status of children: HAZ and body-mass-index-for-age Z-scores (BMIz) in children were calculated according to the WHO guidelines [35] using the WHO Anthro software (version 3.2.2, January 2011). All children <5 y from eligible households were classified as stunted (HAZ < −2 SD) versus not stunted [9,10], and as overweight/obese (BMIz > 2 SD) versus not overweight/obese [35]. BMIz scores were used because they are well suited for statistical analysis and provide a useful interpretation as well as a basis for international reference and comparison [37].

Nutritional status of mother: Body Mass Index (BMI) was calculated and classified using the following WHO cutoff points [38]: underweight (BMI < 18.5 kg/m^2^), normal (BMI 18.5 – 24.9 kg/m^2^), and overweight/obese (BMI ≥ 25 kg/m^2^).

Taking into account all possible combinations of the anthropometric indicators used in this study, nineteen different typologies were identified (Table 2). However, despite finding nineteen possible combinations of anthropometric typologies, most of the typologies were demonstrated by a few prevalent situations including: undernourished HHs, represented by a normal mother & at least one stunted child with the remaining children normal; overweight/obese HHs, represented by an overweight/obese mother and all children normal; and dual burden, represented by an overweight/obese mother and at least one stunted child with the remaining children normal. Typologies were merged into others when it was deemed appropriate based on anthropometric indicators (HAZ, BMIz and BMI) and taking into account potential clustering of risk factors that could influence the family. For example, homes where the mother was overweight/obese and the children were normal were merged with the overweight/obese typology. Homes where the mother was underweight and the children were normal were merged with the undernourished category. Homes were the mother was underweight and there were either stunted and obese children in the same household were merged with the dual burden typology, as stated by final category in Table 2.

**Table 2:** Households anthropometric typologies* identified by year: 2000 (n = 2,876 HHs), 2005 (n = 8,598 HHs), and 2010 (n = 11,349 HHs) (DHS/(ENDS/ENSIN Colombia)

**Household anthropometric typology**	**2000 n (%) 95% C.I**	**2005 n (%) 95% C.I**	**2010 n (%) 95% C.I**	**Final category**
Normal mother & all children normal (neither stunted nor underweight or obese)	1,182 (41.1) 39.2 - 43	3,574 (41.9) 40.4 – 43.3	4,301 (39.3) 38.1 – 40.4	Normal
Underweight mother & all children stunted	0 (0)	0 (0)	0 (0)	Undernourished
Underweight mother & all children normal	62 (2.0) 1.4 – 2.5	283 (3.4) 2.9 – 3.8	312 (2.9) 2.5 – 3.3.	Undernourished
Underweight mother & at least one stunted child the rest are normal	22 (0.1) 0.0 – 0.1	74 (0.07) 0.0 – 0.1	76 (0.1) 0.0 – 0.1	Undernourished
Normal mother & all children stunted	0 (0)	0 (0)	0 (0)	Undernourished
Normal mother & at least one stunted child the remaining are normal	310 (11) 9.6 - 12	801 (9.3) 8.5 – 10.1	862 (6.6) 5.9 – 7.2	Undernourished
Overweight/obese mother & all children normal	932 (32.6) 30.7 – 34.4	2,882 (32.8) 31.4 – 34.2	4,494 (40.0) 38.4 – 40.7	Overweight/obese
Overweight/obese mother & all children overweight/obese	0 (0)	0 (0)	0 (0)	Overweight/obese
Overweight/obese mother & at least one child overweight/obese, the remaining are normal	90 (3.1) 2.4 – 3.7	229 (3.0) 2.2 – 3.1	379 (3.3) 2.8 – 3.7	Overweight/obese
Normal mother & all children overweight/obese	0 (0)	0 (0)	0 (0)	Overweight/obese
Normal mother & at least one child overweight/obese, the remaining are normal	68 (2.4) 1.8 – 3.0	178 (2.4) 1.9 – 2.8	212 (2) 1.7 – 2.3	Overweight/obese
Overweight/obese mother & all children stunted	0 (0)	0 (0)	0 (0)	Dual burden
Overweight/obese mother & at least one stunted child the remaining are normal	196 (6.6) 5.5 – 7.6	544 (6.4) 5.7 – 7.1	660 (5.1) 4.6 – 5.6	Dual burden
Overweight/obese mother & all children stunted and overweight/obese	0 (0)	0 (0)	0 (0)	Dual burden
Overweight/obese mother & at least one child stunted and overweight/obese, the remaining are normal	12 (0.4) 0.0 – 0.6	23 (0.0)	47 (0.0)	Dual burden
Underweight mother & all children overweight/obese	0 (0)	0 (0)	0 (0)	Dual burden
Underweight mother & at least one child overweight/obese, the rest are normal	2 (0)	9 (0.0)	6 (0.0)	Dual burden
Underweight mother & all children stunted and overweight/obese	0 (0)	0 (0)	0 (0)	Dual burden
Underweight mother & at least one child stunted and overweight/obese, the rest are normal	0 (0)	1 (0)	0 (0)	Dual burden

Normal children are defined as neither stunted nor underweight or overweight/obese.

*(Mutually exclusive categories).

### Household anthropometric typologies

Normal Households: No stunting or obesity among any of the children and mother with normal BMI.

Undernourished Households: At least one child is stunted (HAZ < −2) and the remaining children are either stunted or normal and mother classified as normal (BMI 18.5 – 24.9 kg/m^2^) or underweight (BMI <18.5 kg/m^2^).

Overweight/obese Households: At least one child is overweight/obese (BMIz > 2SD) and the remaining children are either overweight/obese or normal and mother classified as normal (BMI 18.5 – 24.9 kg/m^2^) or overweight/obese (BMI > 25 kg/m^2^).

Dual Burden Households: At least one child is stunted (HAZ < −2) and the remaining children can be stunted, normal, overweight/obese, or stunted and overweight/obese (BMIz > 2SD) and mother classified as underweight (BMI <18.5 kg/m^2^) or overweight/obese (BMI > 25 kg/m^2^) or at least one child is overweight/obese (BMIz > 2SD) and the remaining children can be either normal, overweight/obese, or stunted and overweight/obese. If the mother was classified as underweight and at least one child was stunted and the remaining children were normal, this household was included in the underweight typology. The only case in which this home was classified as dual burden was if any of the remaining children was overweight/obese or stunted and overweight/obese. The same was applicable for homes in which the mother was overweight/obese and at least one child was overweight/obese; they were only included in the dual burden typology if the remaining children were either stunted or stunted and overweight/obese.

#### Independent variables

Three household-level stratifying variables were used. The first was area of residence, classified as urban versus rural, based on the definition used in the DHS/ENDS protocols [29-33] as follows: a) urban areas are organized in blocks with clear streets and highways; they include capital cities and municipal main cities, whereas, b) rural areas are disperse and non-disperse areas, have sprawling and farming areas with no clearly defined streets, highways or roads, and generally do not have structured water and sewage systems. The second stratifying variable was wealth index (WI), ranging from 1 to 5, where 1 was the poorest and 5 was the richest household [39]. The wealth index proposed by Rutstein and Johnson in 2004 [39] and developed for use in DHS was used in this study. WI assesses the weighted average in the household of a range of assets, such as television, type of flooring, water supply, refrigerator, electricity, radio, television, and domestic servant. The index is estimated using principal components analysis and is a continuous variable. Quintile categories of the index already calculated and included in the survey were used in this analysis. The last stratifying variable used was states of the country [39].

### Data analysis

Data from ENDS/ENSIN 2000, 2005, and 2010 [29-33] were used to calculate the prevalence of household anthropometric typology by strata: WI, area of residence (urban versus rural), and state. For each one of the stratifying variables, 95% confidence intervals were calculated to assess significance of the change in the prevalence of household anthropometric typology among the three years (2000, 2005, 2010).

The expected versus the observed prevalence of the dual burden of malnutrition were calculated for this study following other international studies [40]. To estimate the expected prevalence of the dual burden at the household level, the prevalence of maternal overweight/obesity was multiplied by the prevalence of stunted children and divided by one hundred. Finally, the expected prevalence was compared with the observed prevalence using a chi-square and obtaining a p value deemed significant at the 0.05 level.

GIS Arc-view [41] was used to graphically depict the transition of anthropometric typologies in the country from 2000, 2005 to 2010 using state layers. STATA 12 [42] was used to calculate weighted prevalence of anthropometric typology stratifying by WI, area of residence, and state. All analyses accounted for the complex sampling design and sampling weights using the command svy.

## Results

Overall, prevalence of stunting (HAZ < −2 SD) among children younger than five years was 18.5%, 95% CI: 17.2%-19.2%, 16.3%, 95% CI: 15.3%-17.1%, and 14.9%, 95% CI: 13%-15.6%, in 2000, 2005 and 2010, respectively (data not shown) (p-test for trend <0.05). Prevalence of overweight/obesity (BMIz > 2SD) among children was 5.7%, 95% CI: 5.3%-6.1% in 2000, 4.5%, 95% CI: 3.2%-5.1% in 2005, and 5.2%, 95 CI: 4.3%-6.2% in 2010 (p-test for trend >0.05). Prevalence of underweight (BMI <18.5 kg/m^2^) among mothers ages 18 to 49 years, was 3.9%, 95% CI: 3.5%-4.6%, 4.7%, 95% CI: 4.3%-5.1%, and 3.9%, 95% CI: 3.1%-4.2% in 2000, 2005, and 2010, respectively (p-test for trend >0.05). Meanwhile, prevalence of overweight/obesity among mothers (BMI ≥ 25 kg/m^2^) was 42.1%, 95% CI: 41.2%-43.5%, 41.1% 95% CI: 40%-42.5%, and 46.8%, 95% CI: 45.7%-47% in 2000, 2005 and 2010, respectively (p-test for trend <0.05). Significant differences in the average BMI of mothers were detected (24.8, 95% C.I: 24.6%-24.9% in 2000, vs. 25.3, 95% C.I: 25.2%-25.4% in 2010) (p-test for trend <0.05).

### Prevalence and patterns of household anthropometric typologies

The percentage of homes classified within the overweight/obese typology increased significantly from 2000 (38.2%, 95% C.I.: 36.2%-40.1%) to 2010 (43.1%, 95% C.I.: 41.9%-44.3%) (p-value <0.05) (Additional file 1). Meanwhile the percentage of homes classified as undernourished significantly decreased between 2005 (13.7%, 95% C.I: 12.7%-14.6%) and 2010 (10.6%, 95% C.I: 9.9%-11.4%) (p-value <0.05). A similar pattern was observed for the dual burden typology (6.5%, 95% C.I.: 5.8%-7.3% in 2005 versus 5.1%, 95% C.I.: 4.5%-5.6% in 2010) (p-value <0.05). The observed prevalence of the dual burden in all three years was not significantly different from the expected prevalence (in 2000 7.1% vs 6.6% p value for chi-square 0.98, in 2005 6.5% vs 7.6% p value for chi-square 0.92, and in 2010 5.1% vs. 6.6%) p value for chi-square 0.84.

### Prevalence and trends of household anthropometric typologies by area of residence

The percentage of overweight/obese HHs was significantly higher among urban versus rural areas in 2005 (P < 0.05) (Additional file 2). Although the prevalence of overweight/obese HHs remains higher in urban versus rural areas in all three years, the rate of growth appears to be larger for rural areas. For instance, in 2010 the gap between urban and rural areas for all anthropometric typologies was decreasing: the prevalence of overweight/obese HHs in 2005 was 38.9% for urban and 33.3% for rural areas, and in 2010, the difference narrowed with 43.7% in urban areas versus 41.6% in rural areas. Likewise, a gap reduction was also seen for undernourished HHs (11.3% in urban and 18.9% in rural areas in 2005, versus 9.3% in urban and 14.1% in rural areas in 2010) and dual burden HHs (5% in urban and 9.9% in rural areas in 2005 versus 4.6% in urban and 6.1% in rural areas in 2010). Meanwhile, dual burden and undernourished typologies were significantly more prevalent among rural areas in 2000 and 2005 (p < 0.05).

### Prevalence and trends of anthropometric typology households by WI

The prevalence of overweight/obese HHs increased with WI across all three years, but in 2010 the gap between lowest and highest WI was smallest (Additional file 3). For instance, the prevalence of overweight/obese HHs in the lowest wealth quintile was 30.6% versus 43.9% in the highest quintile in 2000; meanwhile, the prevalence of overweight/obese HHs in the lowest wealth quintile was 39.6% versus 49.2% in the highest quintile in 2010. Although the overweight/obesity typology continues to be more prevalent among the fourth and highest wealth quintile in all three years, the rate of increase between 2000 and 2010 is larger among households in the first (9 percentage points (pp)) and second wealth quintile (7.3 pp) compared to the fourth (−0.8 pp) and fifth quintiles (5.3 pp).

The prevalence of undernourished HHs decreased as WI increased (Additional file 4). Similar to the overweight/obese typology, prevalence is becoming more homogenous throughout the years as the gap between the lowest and the highest wealth quintile seems to be narrowing. For example, the prevalence of undernourished HHs in 2005 was 23.4% in the lowest wealth quintile and 4.3% in the highest wealth quintile, versus 15.7% in the lowest wealth quintile and 5.3% in the highest wealth quintile for the year 2010. Nevertheless, there are significant differences in the prevalence of undernourished between the lowest and the highest wealth quintiles, with almost three times higher prevalence among the poorest in 2010.

Similar to the undernourished typology, the prevalence of dual burden households decreased as WI increased (Additional file 5). The differences between wealth quintiles are more homogenous in the year 2010 compared to 2000 and 2005. In particular, there has been a significant reduction in the prevalence of dual burden for the lowest WI between 2005 and 2010.

### State variation in the prevalence of anthropometric typology households

**Figure 1:**
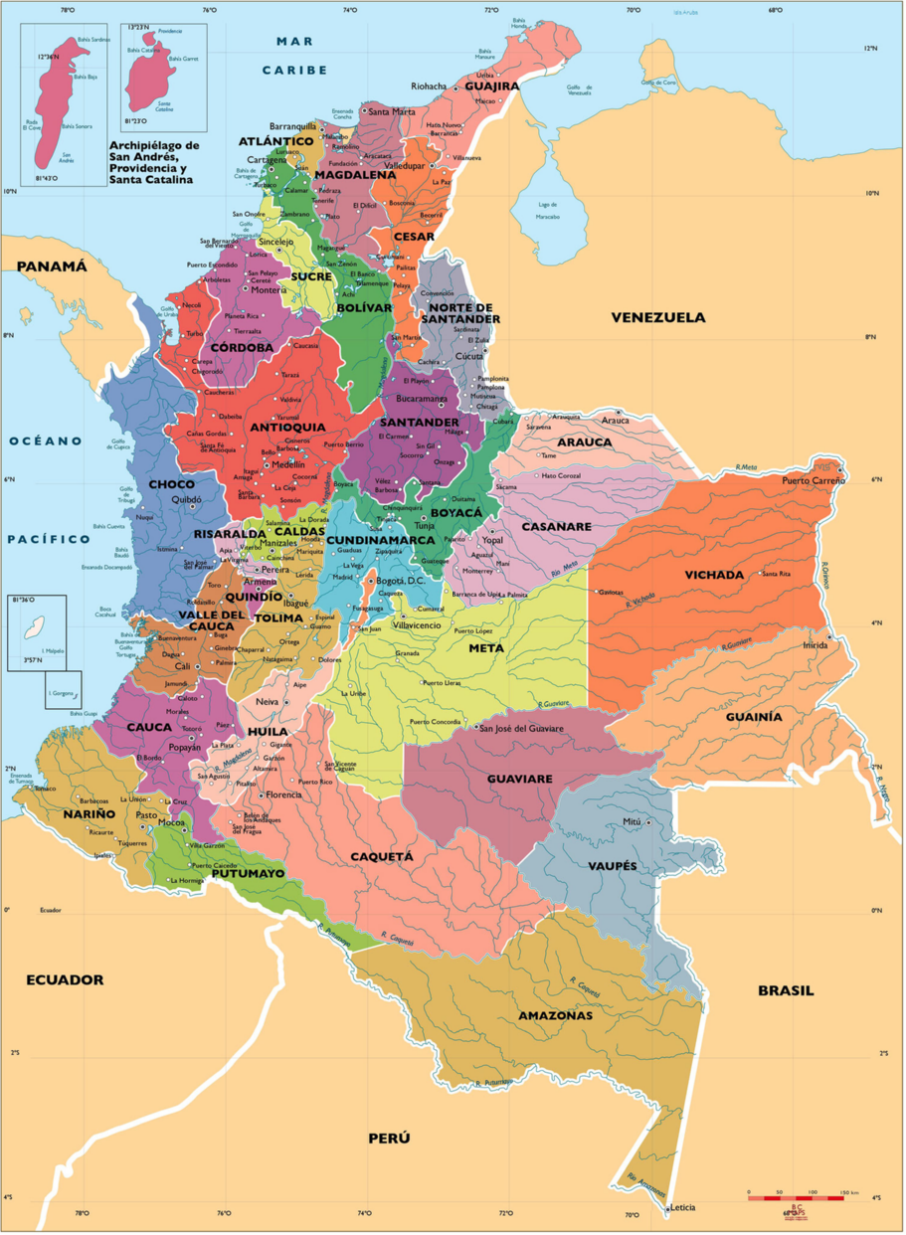
**Map of Colombian States to use as reference for subsequent graphs.**

**Figure 2:**
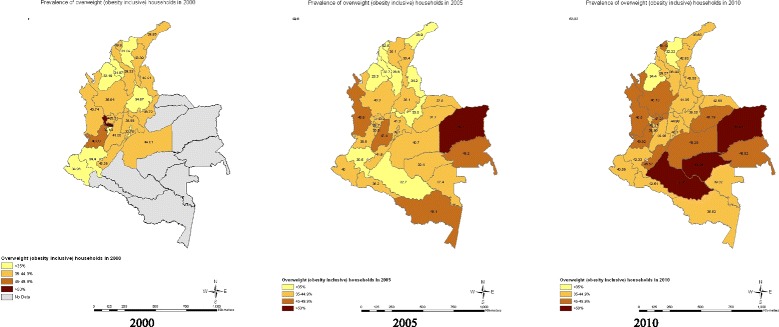
**Prevalence of overweight/obese household anthropometric typology by state in 2000 n = 2,876 HHs, 2005 n = 8,598 HHs, 2010 n = 11,349 HHs (ENDS/ENSIN Colombia).** Note: Grey depicts the regions of San Andrés, Orinoco and Amazonas, which were not included in the sample selection of DHS 2000. Overweight/obese Households: At least one child is overweight/obese (BMIz > 2SD) and the remaining children are either overweight/obese or normal.

**Figure 3:**
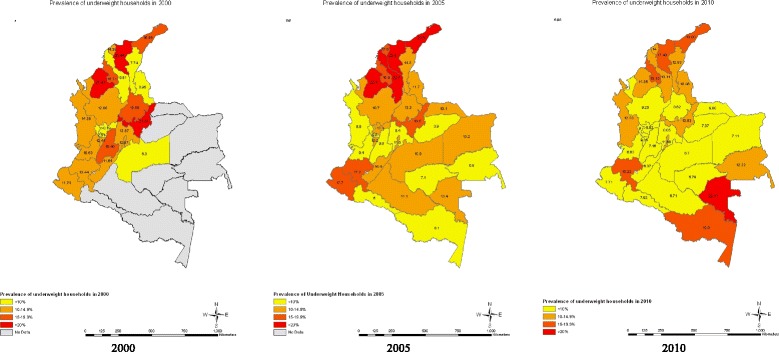
**Prevalence of undernourished household anthropometric typology by state in 2000 n = 2,876 HHs, 2005 n = 8,598 HHs, 2010 n = 11,349 HHs (ENDS/ENSIN Colombia).** Note: Grey depicts the regions of San Andrés, Orinoco and Amazonas, which were not included in the sample selection of DHS 2000. Undernourished Households: At least one child is stunted (HAZ < −2) and the remaining children are either stunted or normal.

**Figure 4:**
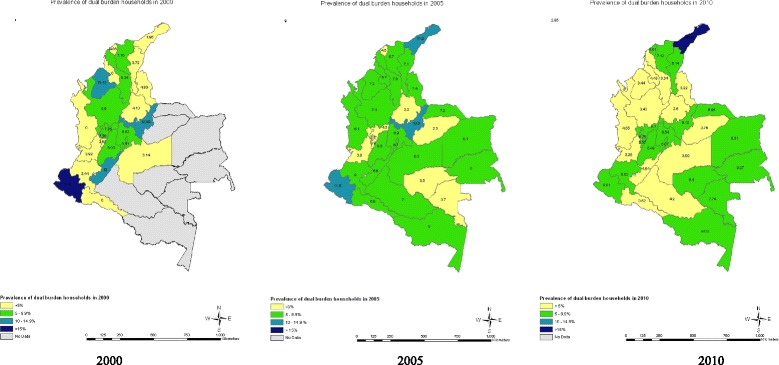
**Prevalence of dual burden household anthropometric typology by state in 2000 n = 2,876 HHs, 2005 n = 8,598 HHs, 2010 n = 11,349 HHs (ENDS/ENSIN Colombia).** Note: Grey depicts the regions of San Andrés, Orinoco and Amazonas, which were not included in the sample selection of DHS 2000. Dual Burden Households: At least one child is stunted (HAZ < −2) and the remaining children can be either stunted, normal, or stunted and overweight/obese OR At least one child is overweight/obese (BMIz > 2SD) and the remaining children can be either normal, overweight/obese, or stunted and overweight/obese.

Within state variations across time are depicted in maps for overweight/obese (Figure 1, Figure 2), undernourished (Figure 3), and dual burden (Figure 4) HHs. The progression of the various anthropometric typologies between 2000, 2005 and 2010 are presented in the maps showing an overall increase of overweight/obese HHs and an overall reduction in the prevalence of undernourished and dual burden HHs. Regarding the overweight/obesity category, almost every state from Colombia experienced an increase in the percentage of overweight/obese households, but the states located in the south-east region saw the highest increase (Figure 2). In contrast, the prevalence of undernourished households decrease overtime across all states of Colombia, with the exception of the southern states of Amazonas and Vaupes, which actually saw an increase (Figure 3). Regarding the dual burden of malnutrition, all of the states of Colombia experienced a decrease in the prevalence overtime with the exception of the Northern state of la Guajira (Figure 4). For reference of the location of each state please refer to Figure 1.

## Discussion

This study is among the first to document the nutrition transition in Colombia over a decade using objectively measured anthropometric data from large nationally representative surveys. In addition, this study developed a novel classification system that considers all children in the household as well as their mother. This approach also allows identifying a double burden of malnutrition within a household, where one child may be overweight and another one undernourished. The current study provides important information regarding the nutrition transition in Colombia including the prevalence and extent of the dual burden of malnutrition at the state level and not only at the national or regional level [10]. Identifying the multiple typologies within households provides for the first time a diagnosis of the nutritional status of the dyads of children and mothers. Results from this study suggest that the changes in the nutrition transition in Colombia over the 10 y period are mainly demonstrated by two factors - increasing overweight/obesity in mothers and decreasing stunting in children. This pattern was confirmed by a decrease in the percentage of the population in the normal weight category and a shift towards the overweight/obese typology. Similar trends including the decrease in the dual burden have been reported in most countries from Latin America and the Caribbean undergoing a nutrition transition [43-50].

The prevalence of overweight/obese households was higher among high WI versus middle and lower WI households. However, there was a larger increase over time among lowest WI categories compared to the highest WI categories. This indicates that although overweight/obesity continues to be more prevalent among high-income populations, it is growing at a faster rate among the most economically disadvantaged as seen in other countries from Latin America including Brazil and Mexico [21-23]. The observed prevalence of dual burden across all three years in the Colombia surveys did not differ significantly from the expected prevalence [40]. However, states like La Guajira need closer attention, since it was the only state in which the prevalence of dual burden increased over time which may be related to the confluence of adverse socioeconomic conditions, public utility deficiencies, and political corruption [51,52], as well as a high prevalence of indigenous populations whose high prevalence of dual burden has been reported elsewhere [53-56].

This study found important state disparities of various anthropometric typologies for overweight/obese, undernourished and dual burden HHs. Regional differences must be considered when designing and implementing nutrition policies at the population level. For example, the Pacific region (i.e. Chocó and Cauca states) is marked by poverty and inequalities, as well the effects of a long lasting armed internal conflict which is higher than the burden experienced from these problems in other regions of the country [57]. Effects of the internal conflict that directly affect the nutritional status of the population are related to shortages of food supply and food security and have been documented in several reports by national and international agencies [58,59]. More than 90% of the population of the Pacific region is of African descent, which has been traditionally marginalized, undermined, and discriminated in the country, similarly to the indigenous population. This population has been subjected to conditions of poverty over many generations, which have directly affected their nutritional status [60]. The region occupies regular headlines in Colombian newspapers as the area with one of the worst prevalences of child undernutrition in the country [57]. However, evidence from the present study shows that the region is also suffering from a concomitant increase of overweight/obesity, in this case both problems could potentially be linked to conditions of poverty such as lack of access to basic sanitation, clean water and infrastructure, which have traditionally been associated with undernourishment [61], but also possibly to easy access and higher consumption of energy dense nutrient poor foods [62]. Higher or normal levels of BMI do not necessarily translate to good nutrition; in fact many micronutrient deficiencies can still exist even under conditions of overweight and obesity [63,64]. Recent studies, mainly from low and middle-income countries have documented the coexistence of anemia and obesity among the adult [65,66] and child population [67]. According to the last Colombian nutrition survey in 2010, the Pacific region has one of the highest rates of anemia in the country with 32.2% among children 6 to 59 months, and 13% among women 13 to 49 years old, compared to the national average of 27.7% and 7.6%, respectively [7]. The maps produced in this paper highlight regional inequalities in the distribution of malnutrition problems in the country, in particular for the undernourished and dual burden typologies, which are higher in states with lower human development indexes and higher prevalence of unmet basic needs such as Amazonas and Orinoco. Further analysis need to be conducted to disentangle mechanisms of racial health inequalities with respect to underweight and dual burden households.

Some limitations of this study deserve mention. First, the use of secondary data brings the usual problems associated with this type of data, including the inability to obtain additional information directly from the respondents. Much of the information used in this study was self-reported and could be affected by social desirability bias [68]. Other types of measurement error could exist for anthropometric measures, however standardized international training was provided to the interviewers for all years of ENDS/ENSIN (2000, 2005, and 2010) and the likelihood of errors is minimal. A different sampling frame was used in 2000, by excluding the rural regions of San Andrés, Orinoco and Amazonas, which limits the reach of the conclusions. Finally, although the outcome variable created for this study offers a novel and comprehensive approach, it combines information from various anthropometric categories, which could have reduced the ability to detect true differences. Nonetheless, as mentioned earlier, the final four categories (normal, overweight/obesity, undernourished, dual burden HHs) are driven only by a few prevalent anthropometric typologies.

Strengths of this study include the use of large national nutrition surveys that span a decade, providing rich information that allows producing a nutritional profile of the Colombian population through a period of profound economic, political and social changes. In addition, the new household classification system of anthropometric typologies developed in this study is an advancement over traditional methods since it allows the inclusion of all the children in the household and also of concomitant types of malnutrition including chronic undernutrition, overweight/obesity and dual burden at the individual level. This is the major innovation of the study and can have vast potential for assessing the population at the national level and for better targeting nutrition interventions and policies. Using information from publicly available data from Demographic and Health Surveys, it is possible for other countries from Latin America to assess the household level prevalence of malnutrition using this novel classification system. Doing so would allow for comparative studies throughout the region and establishing the prevalence of the dual burden of malnutrition at the household level, a phenomenon that still needs to be assessed and explored in some countries from the region.

## Conclusion

Colombia is in the midst of a nutrition transition that is mirroring the changes occurring elsewhere in the world, such as an increase in the overweight/obese population and a general decrease in its chronically undernourished population. In general, some homogeneity in the distribution of anthropometric typologies by WI and area of residence (urban versus rural) was observed in this study. Although overweight/obesity continues to be more prevalent in urban versus rural areas, the rate of growth is higher among rural versus urban populations. Similar trends are seen for WI. While overweight/obesity increased with WI across all three years, a higher increase of overweight was observed for the lowest category of WI. A concerning finding documented in this study are the large regional inequalities in the prevalence of undernourished and dual burden HHs (Additional file 6: Graph 1, 3), and should be addressed by national, state, and local policies and programs that focus on increasing diet quality and promoting physical activity. The forthcoming 2015 national nutritional survey from Colombia will include the estimation of the dual burden at the household level in part based on the work developed in this study and similar efforts [69,70]. Although Colombia does not seem to have a prevalence of the dual burden of malnutrition beyond what would be normally expected based on the national prevalence [40], it is important for the food security policy of Colombia to consider the possibility of this phenomenon at the household level.

## Additional files

Additional file 1:
**Household anthropometric typologies* by year 2000 n=2,876 HHs, 2005 n= 8,598 HHs, and 2010 n=11,349 HHs (ENDS/ENSIN Colombia).**
Additional file 2:
**Household anthropometric typologies by area of residence (urban versus rural) in 2000 n=2,876 HHs, 2005 n= 8,598 HHs, 2010 n=11,349 HHs (ENDS/ENSIN Colombia).**
Additional file 3:
**Overweight/obese household anthropometric typology by WI in 2000 n=2,876 HHs, 2005 n= 8,598 HHs, 2010 n=11,349 HHs (ENDS/ENSIN Colombia).**
Additional file 4:
**Undernourished household anthropometric typology by WI in2000 n=2,876 HHs, 2005 n= 8,598 HHs, 2010 n=11,349 HHs (ENDS/ENSIN Colombia).**
Additional file 5:
**Dual Burden household anthropometric typology by WI in 2000 n=2,876 HHs, 2005 n= 8,598 HHs, 2010 n=11,349HHs (ENDS/ENSIN Colombia).**
Additional file 6:
**Graph 1.** Prevalence of overweight/obese household anthropometric typology by state in 2000 n=2,876 HHs, 2005 n= 8,598 HHs, 2010 n=11,349 HHs (ENDS/ENSIN Colombia). Graph 2. Prevalence of undernourished household anthropometric typology by state in 2000 n=2,876 HHs, 2005 n= 8,598 HHs, 2010 n=11,349 HHs (ENDS/ENSIN Colombia). Graph 3. Prevalence of dual burden household anthropometric typology by state in 2000 n=2,876 HHs, 2005 n= 8,598 HHs, 2010 n=11,349 HHs (ENDS/ENSIN Colombia).
