# Supplementary material for: The nutrition transition in Colombia over a decade: a novel household classification system of anthropometric measures
Source: Arch Public Health. 2015 Feb 16;73(1):12. doi: 10.1186/s13690-014-0057-5 (PMC4361151; doi:10.1186/s13690-014-0057-5)
Supplement: Additional file 1: — Household anthropometric typologies* by year 2000 n=2,876 HHs, 2005 n= 8,598 HHs, and 2010 n=11,349 HHs (ENDS/ENSIN Colombia). [file 13690_2014_57_MOESM1_ESM.docx]

Additional file 1. *Household anthropometric typologies* by year 2000 n=2,876 HHs, 2005 n= 8,598 HHs, and 2010 n=11,349 HHs (ENDS/ENSIN Colombia)*

*

*

*

*

*

*

Overweight/obese Undernourished Dual Burden

* Data are statistically different between 200 and 2010 or 2005 and 2010 based on no overlap of 95% confidence intervals.

*Overweight/obese Households:* At least one child is overweight/obese (BMIz>2SD) and the remaining children are either overweight/obese or normal

*Undernourished Households:* At least one child is stunted (HAZ<-2) and the remaining children are either stunted or normal

*Dual Burden Households*: At least one child is stunted (HAZ<-2) and the remaining children can be either stunted, normal, or stunted and overweight/obese OR At least one child is overweight/obese (BMIz>2SD) and the remaining children can be either normal, overweight/obese, or stunted and overweight/obese
