# Supplementary material for: The nutrition transition in Colombia over a decade: a novel household classification system of anthropometric measures
Source: Arch Public Health. 2015 Feb 16;73(1):12. doi: 10.1186/s13690-014-0057-5 (PMC4361151; doi:10.1186/s13690-014-0057-5)
Supplement: Additional file 4: — Undernourished household anthropometric typology by WI in2000 n=2,876 HHs, 2005 n= 8,598 HHs, 2010 n=11,349 HHs (ENDS/ENSIN Colombia). [file 13690_2014_57_MOESM4_ESM.docx]

Additional file 4. Undernourished household anthropometric typology by WI in2000 n=2,876 HHs, 2005 n= 8,598 HHs, 2010 n=11,349 HHs (ENDS/ENSIN Colombia)

*

*

2000 2005 2010

* Data are statistically different between first and second WI in 2005 and between fourth and fifth WI based on no overlap of 95% confidence intervals.

*Undernourished Households:* At least one child is stunted (HAZ<-2) and the remaining children are either stunted or normal

*
