# Supplementary material for: The nutrition transition in Colombia over a decade: a novel household classification system of anthropometric measures
Source: Arch Public Health. 2015 Feb 16;73(1):12. doi: 10.1186/s13690-014-0057-5 (PMC4361151; doi:10.1186/s13690-014-0057-5)
Supplement: Additional file 6: — Graph 1. Prevalence of overweight/obese household anthropometric typology by state in 2000 n=2,876 HHs, 2005 n= 8,598 HHs, 2010 n=11,349 HHs (ENDS/ENSIN Colombia). Graph 2. Prevalence of undernourished household anthropometric typology by state in 2000 n=2,876 HHs, 2005 n= 8,598 HHs, 2010 n=11,349 HHs (ENDS/ENSIN Colombia). Graph 3. Prevalence of dual burden household anthropometric typology by state in 2000 n=2,876 HHs, 2005 n= 8,598 HHs, 2010 n=11,349 HHs (ENDS/ENSIN Colombia). [file 13690_2014_57_MOESM6_ESM.docx]

Additional file 6

Graph 1. Prevalence of overweight/obese household anthropometric typology by state in 2000 n=2,876 HHs, 2005 n= 8,598 HHs,

2010 n=11,349 HHs (ENDS/ENSIN Colombia)


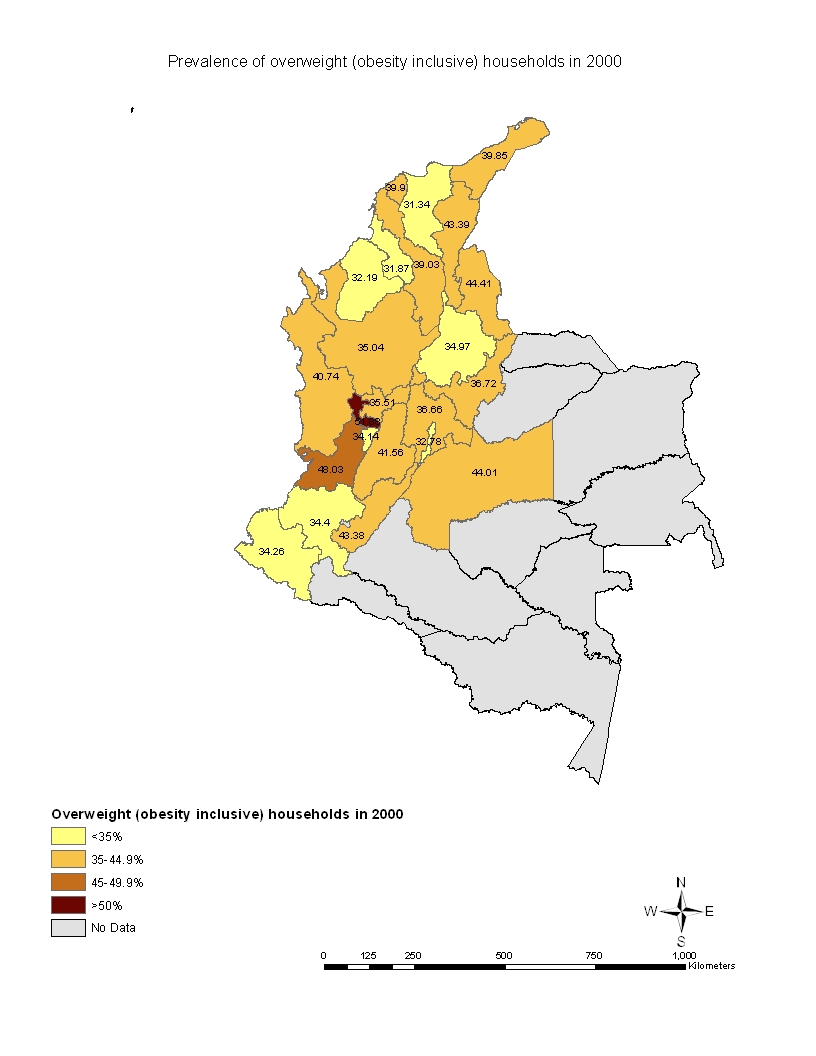

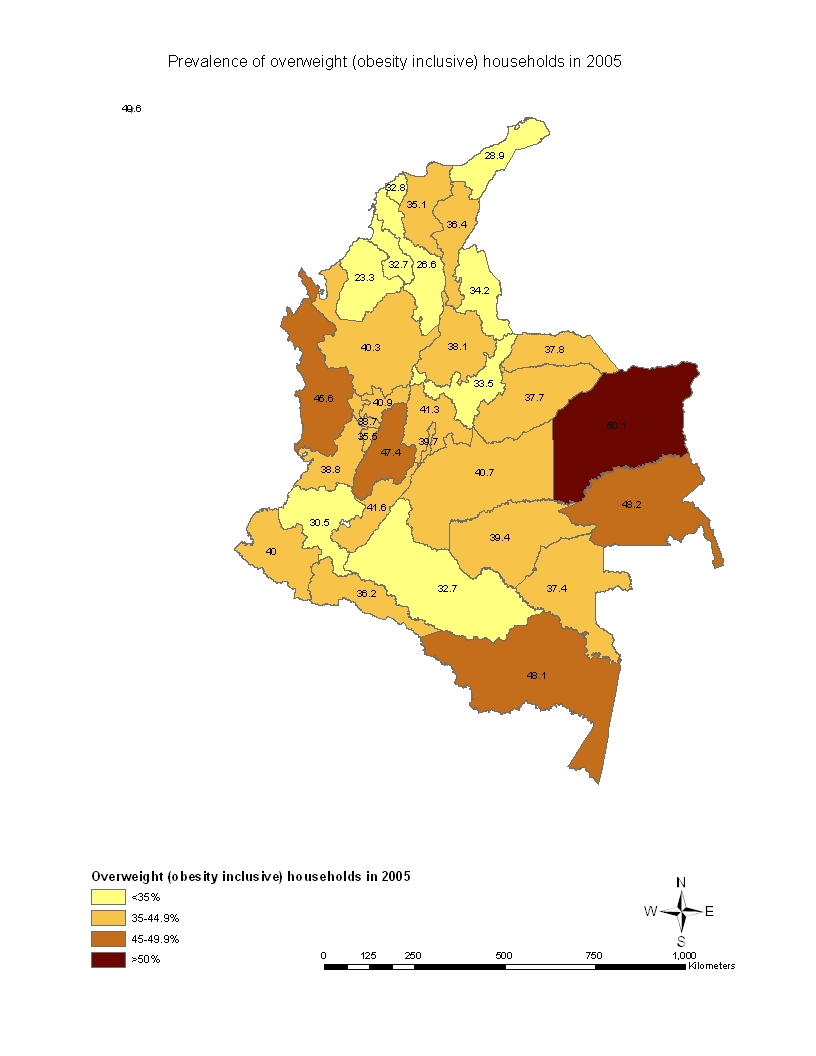

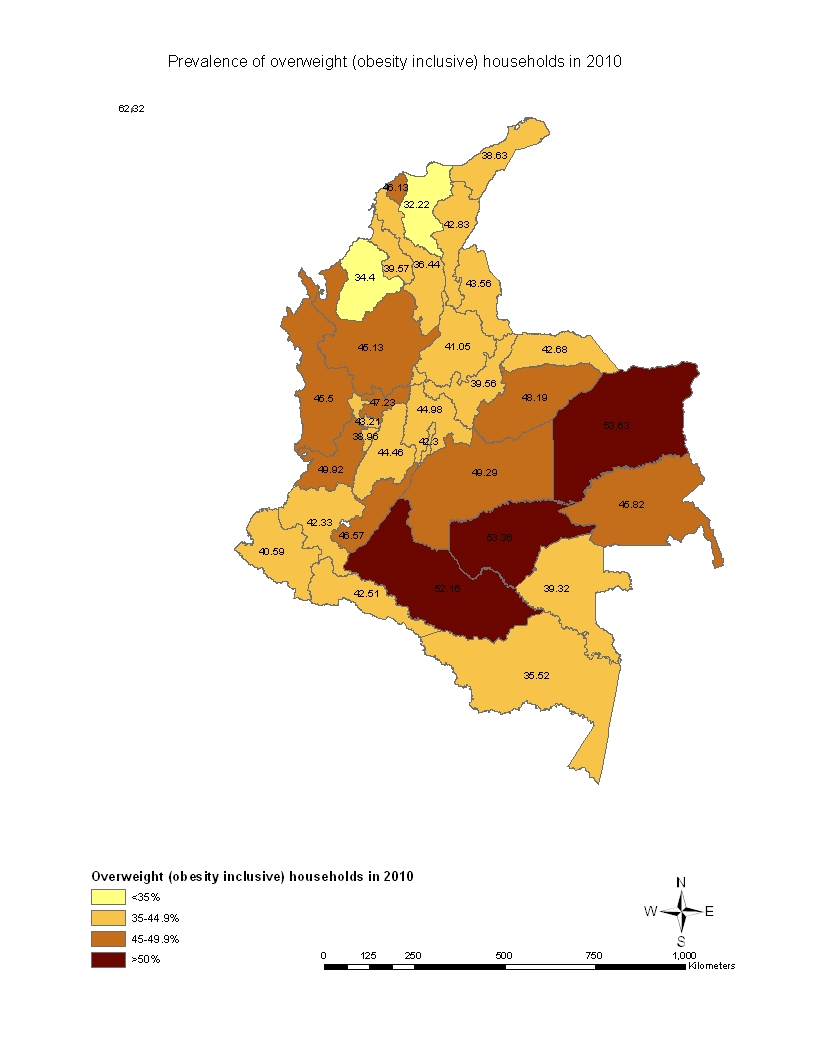


2000

2010

2005

Note: Grey depicts the regions of San Andrés, Orinoco and Amazonas, which were not included in the sample selection of DHS 2000

*Overweight/obese Households:* At least one child is overweight/obese (BMIz>2SD) and the remaining children are either overweight/obese or normal

*

Graph 2. Prevalence of undernourished household anthropometric typology by state in 2000 n=2,876 HHs, 2005 n= 8,598 HHs,

2010 n=11,349 HHs (ENDS/ENSIN Colombia)


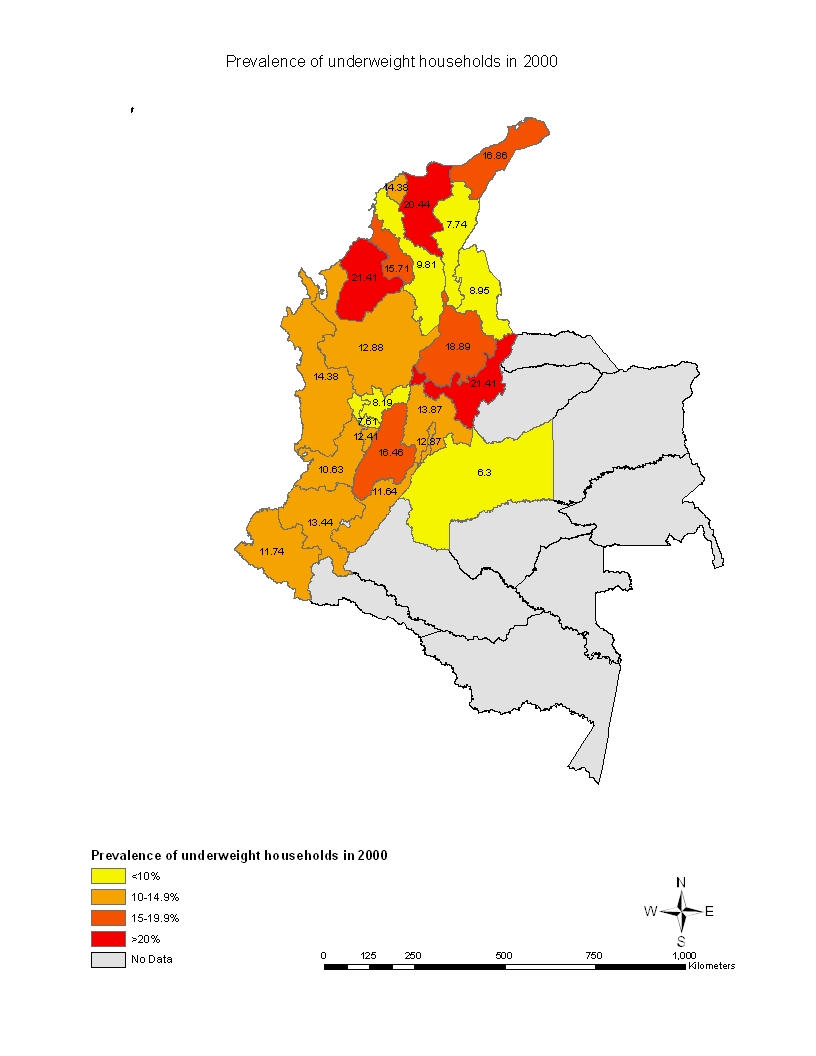

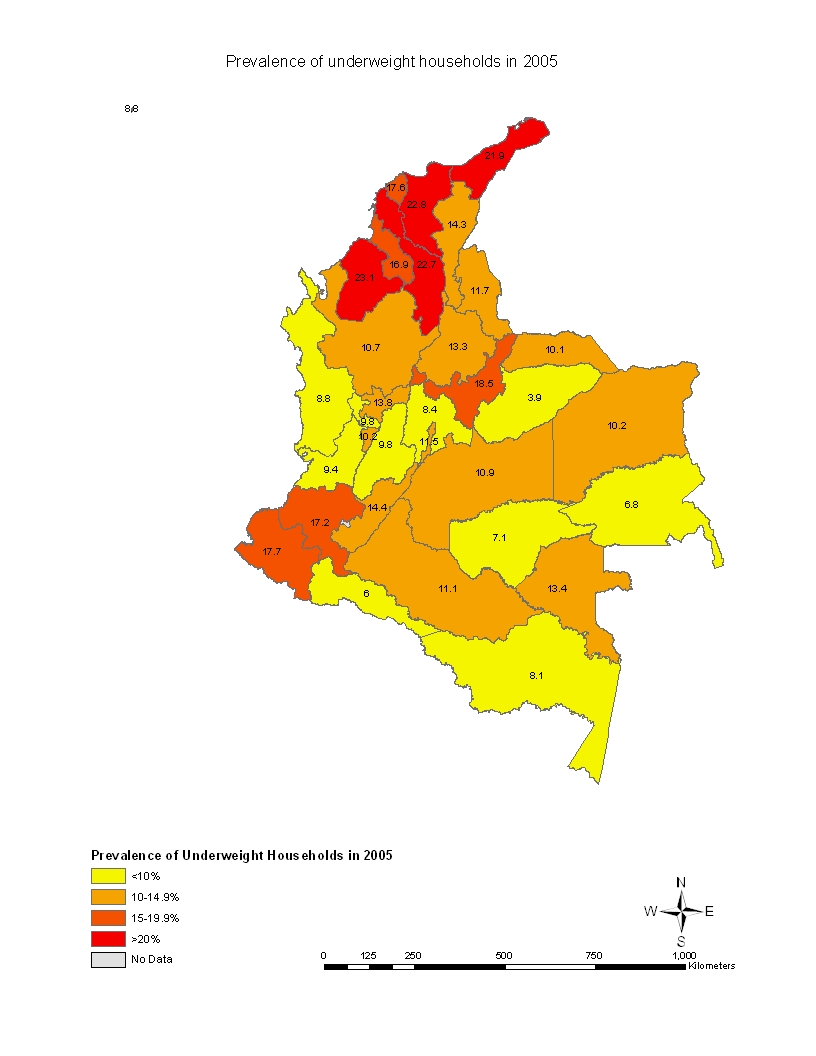

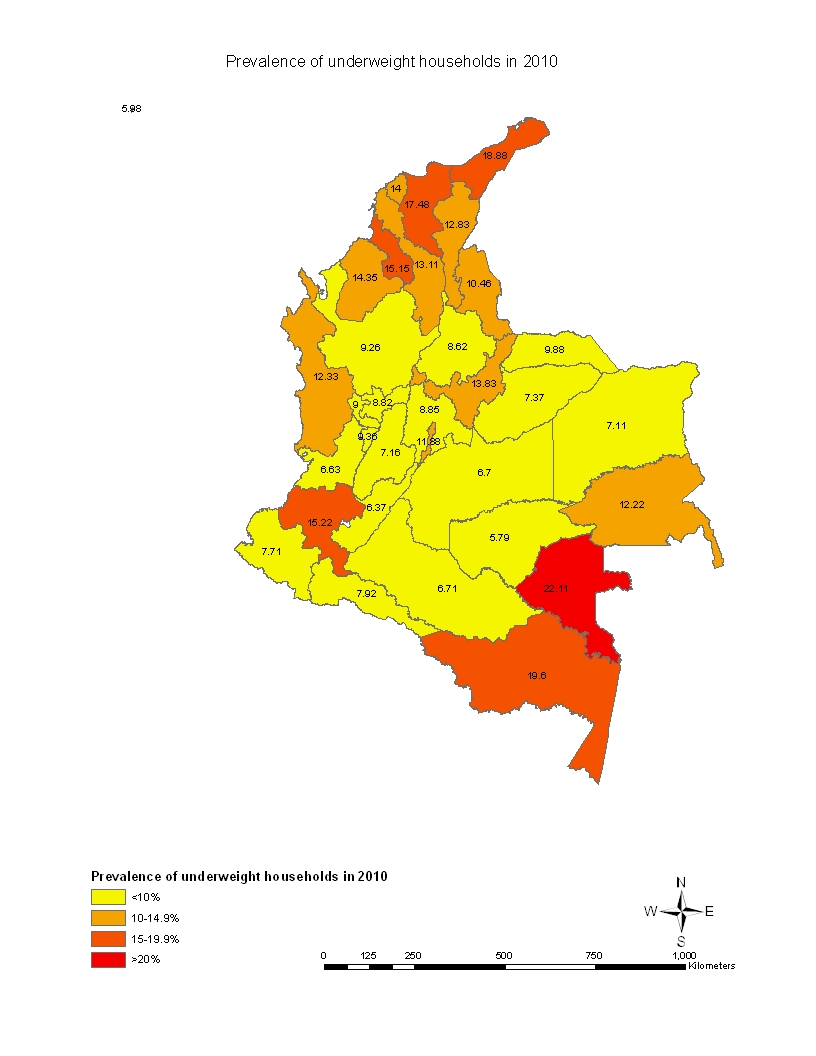


2010

2005

2000

Note: Grey depicts the regions of San Andrés, Orinoco and Amazonas, which were not included in the sample selection of DHS 2000

Undernourished Households: At least one child is stunted (HAZ<-2) and the remaining children are either stunted or normal

Graph 3. Prevalence of dual burden household anthropometric typology by state in 2000 n=2,876 HHs, 2005 n= 8,598 HHs,

2010 n=11,349 HHs (ENDS/ENSIN Colombia)


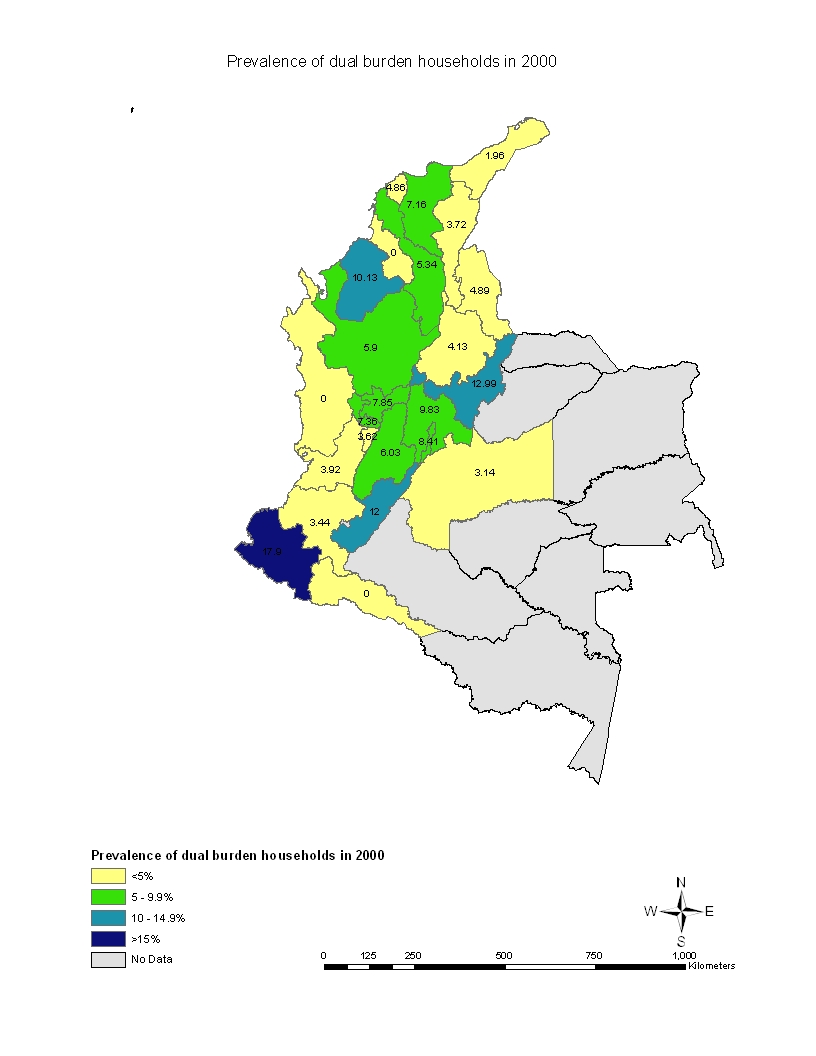

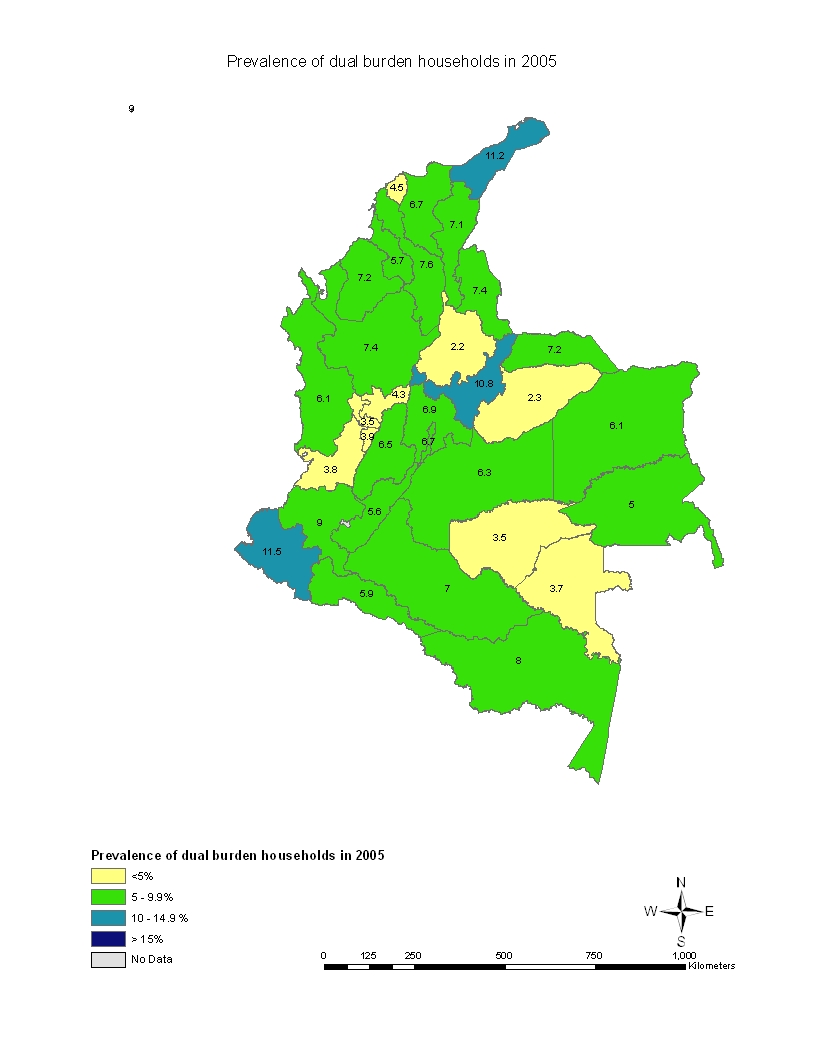

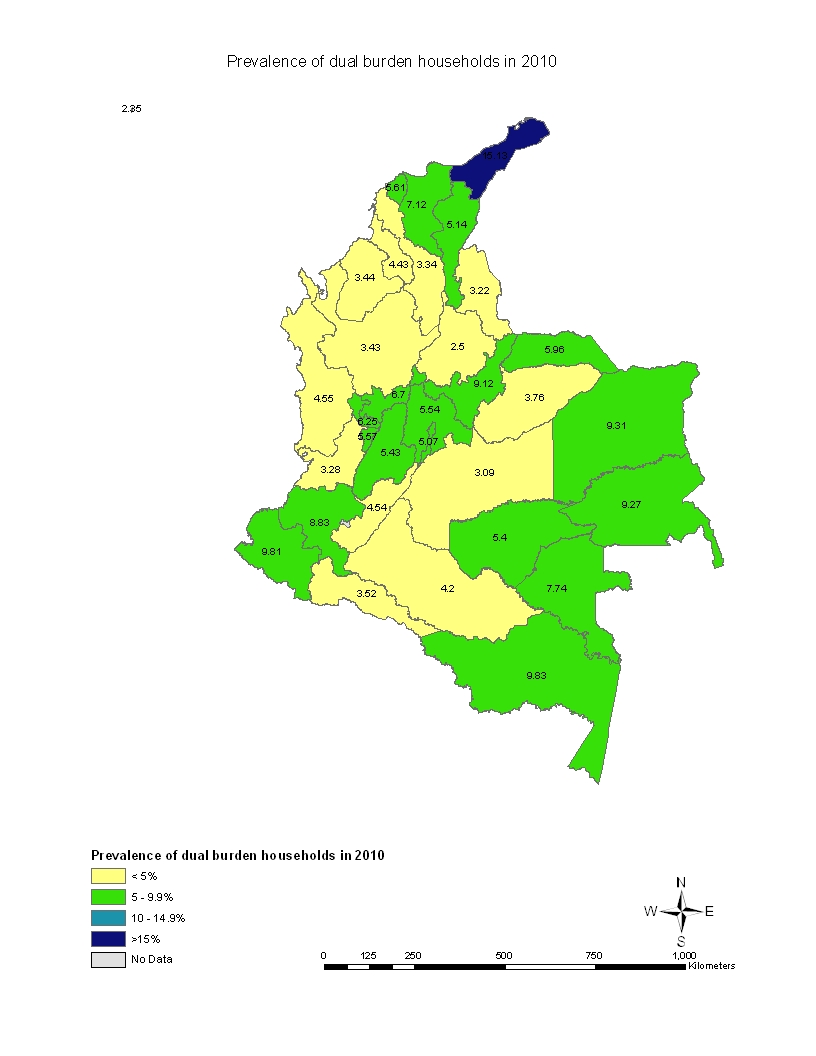


2010

2005

2000

Note: Grey depicts the regions of San Andrés, Orinoco and Amazonas, which were not included in the sample selection of DHS 2000

*Dual Burden Households:* At least one child is stunted (HAZ<-2) and the remaining children can be either stunted, normal, or stunted and overweight/obese OR At least one child is overweight/obese (BMIz>2SD) and the remaining children can be either normal, overweight/obese, or stunted and overweight/obese
